# Efficient haplotype matching and storage using the positional Burrows–Wheeler transform (PBWT)

**DOI:** 10.1093/bioinformatics/btu014

**Published:** 2014-01-09

**Authors:** Richard Durbin

**Affiliations:** Wellcome Trust Sanger Institute, Wellcome Trust Genome Campus, Cambridge CB10 1SA, UK

## Abstract

**Motivation:** Over the last few years, methods based on suffix arrays using the Burrows–Wheeler Transform have been widely used for DNA sequence read matching and assembly. These provide very fast search algorithms, linear in the search pattern size, on a highly compressible representation of the dataset being searched. Meanwhile, algorithmic development for genotype data has concentrated on statistical methods for phasing and imputation, based on probabilistic matching to hidden Markov model representations of the reference data, which while powerful are much less computationally efficient. Here a theory of haplotype matching using suffix array ideas is developed, which should scale too much larger datasets than those currently handled by genotype algorithms.

**Results:** Given *M* sequences with *N* bi-allelic variable sites, an *O*(*NM*) algorithm to derive a representation of the data based on positional prefix arrays is given, which is termed the positional Burrows–Wheeler transform (PBWT). On large datasets this compresses with run-length encoding by more than a factor of a hundred smaller than using gzip on the raw data. Using this representation a method is given to find all maximal haplotype matches within the set in *O*(*NM*) time rather than *O*(*NM*^2^) as expected from naive pairwise comparison, and also a fast algorithm, empirically independent of *M* given sufficient memory for indexes, to find maximal matches between a new sequence and the set. The discussion includes some proposals about how these approaches could be used for imputation and phasing.

**Availability:** http://github.com/richarddurbin/pbwt

**Contact:** richard.durbin@sanger.ac.uk

## 1 INTRODUCTION

Given a large collection of aligned genetic sequences, or haplotypes, it is often of interest to find long matches between sequences within the collection, or between a new test sequence and sequences from the collection. For example, sufficiently long identical substrings are candidates to be regions that are identical by descent (IBD) from a common ancestor. (I will use the word ‘substring’ to denote contiguous subsequences, as is standard in the computer science text matching literature.) When using imputation approaches to infer missing values one wants to identify sequences that are as close as possible to the test sequence around the location being imputed, such as those that are IBD, or at least share long matches with the test sequence. Maximizing the number of such long matches could also form the basis of genotype phasing.

Naive substring match testing would take 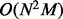 time for each test sequence, where there are *N* variable sites and *M* sequences, and hence 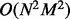 time for complete all-pairs comparison within a set of sequences. By keeping a running match score to find maximal matches as in BLAST, it is straightforwardly possible to reduce this to *O*(*NM*) per single test, and so 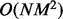 across the whole collection, but this is still large for large *M*. Recently suffix-array-based methods have proved powerful in standard sequence matching, as exemplified by Bowtie (Langmead *et al.*, 2009), BWA (Li and Durbin, 2009) and SOAP2 (Li *et al.*, 2009). Here an approach based on suffix arrays is described that can find best matches within a set of sequences in *O*(*NM*) time, following preprocessing of the dataset also in *O*(*NM*) time, and empirically best single haplotype matches in *O*(*N*) time.

The differences between the algorithms described here and standard suffix array based sequence matching are derived from the fact that there are many sequences that are all of the same length and already aligned. So on the one hand there is no need to consider offsets of the test sequence with respect to the sequences in the collection, but on the other hand the test sequence is long and we are looking for maximal matches of an arbitrary substring of the test sequence, not of the whole test sequence.

## 2 APPROACH

When looking at genetic data from humans or other diploid organisms, there are two underlying genome sequences per person, one from their father and one from their mother. These are known as ‘haplotype’ sequences. Here I consider the case where we are given these two sequences separately, rather than unphased diploid ‘genotype’ sequences, where the two haplotype sequences have been observed together.

Consider a set *X* of *M* haplotype sequences 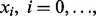 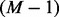 over *N* variable sites indexed by *k*, numbered from 0 to (*N* − 1). We can take all the sites to be bi-allelic with values 0 or 1, so a typical site 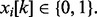 For any sequence *s*, let us write 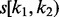 to represent the semi-open substring of *s* starting at *k*_1_ and finishing at 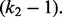 We will say that there is a ‘match’ between *s* and *t* from *k*_1_ to *k*_2_ if 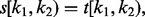 and this match is ‘locally maximal’ if there is no extension that is also a match, i.e. if 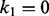 or 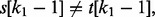 and 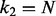 or 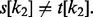 When comparing *s* to the set of sequences *X* we say that *s* has a *set-maximal* match to *x_i_* from *k*_1_ to *k*_2_ if the match is locally maximal and there is no longer match from *s* to any other *x_j_* that includes the interval 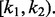 For some applications we will be interested in the set-maximal matches within *X*, i.e. the set-maximal matches of each *x_i_* to 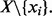


Fundamental to our approach will be to consider a particular form of ordering on substrings of the set *X* of sequences. Here is an explanation for this ordering, with some motivation about why it is important. We will consider a separate ordering for each position *k* between 0 and *N*. For given *k*, let us order the sequences *x* in *X* so that their reversed prefixes 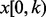 are ordered, by which I mean that the reversed sequences of the prefixes running back from (*k* − 1) to 0 are ordered in the natural fashion, with them being ordered according to their index *i* in *X* if the prefixes are the same.

Let us consider a set-maximal match between two sequences in *X* from *k′* to *k*. If we sort in this reversed order at *k*, then the maximally matching sequences must be adjacent, because, if there were another prefix sorting in between them, then it would have to match both from *k′* to *k* because of sort order, and it would have to match one of the two at position 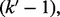 because the maximally matching sequences must take different values there and there are only two possible values. The new sequence would thus form a longer match, which contradicts the presumption that the match between the original pair was set-maximal. Strictly, this argument requires that the original match was set-maximal in both directions. We will see this is important below. However, this is just a motivating paragraph so there is no need to consider yet the case where it is set-maximal in only one direction.

Those with prior exposure to suffix array algorithms will notice that I talk here about prefixes and reversed ordering rather than suffixes and lexicographic ordering. In this text the direction of the standard theory is reversed so that algorithms process forwards naturally through the sequences from 0 to (*N* − 1), rather than backwards; this has no substantive effect on any of the algorithms or results, but will enable us to process very large datasets in the order in which they naturally come, and also makes some of the notation more natural.

### 2.1 Derivation of prefix array representation

**Fig. 1.**
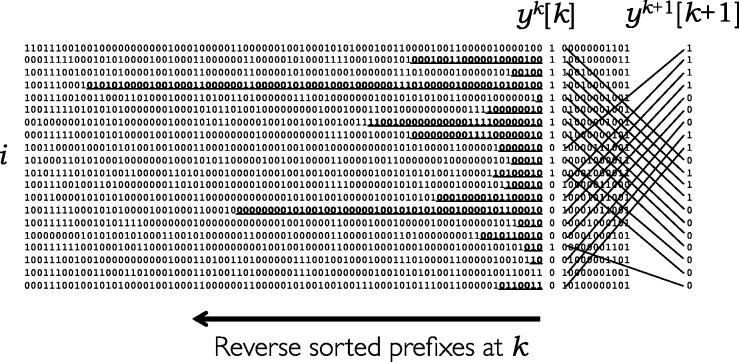
A set of haplotype sequences sorted in order of reversed prefixes at position *k*, showing the set of values at *k* isolated from those before and after, and on the right hand side how the order at position (*k* + 1) is derived from that at *k* as in Algorithm 1. Maximal substrings shared with the preceding sequence ending at *k* are shown bold underlined; these start at position *d_k_*[*i*] as calculated in Algorithm 2

The argument in the preceding paragraph suggests that having the sequences sorted in order of reversed prefixes at position *k* would help find maximal matches. It might seem that finding this sort order for all the prefixes at every position *k* would be computationally costly, but that is not the case. If we know the sort order at position *k* Algorithm 1 shows how to derive the sort order at position (*k *+ 1) by a simple process looking only at the *k*-th value of each sequence. We can therefore calculate the entire set of orderings for all *k* in a single pass through all the sequences, in time proportional to *NM*. Let 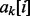 be the index 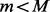 of the sequence *x_m_* from which the *i*-th prefix in the reversed ordering at *k* is derived. The array *a_k_* is a permutation of the numbers 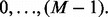 Because we are often going to want to discuss the sequences sorted in the order of their prefixes, I define 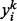 to be the *i*-th sequence in this sorted ordering 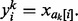 The key observation is that, conditional on the value of 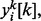 the order of the elements of 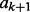 is the same as their order in *a_k_*. An illustration is given in Figure 1.

**Algorithm 1** BuildPrefixArray—build the positional prefix array 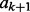 from *a_k_*

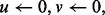
 create empty arrays 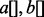

**for** 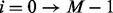 **do**

  **if** 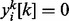 **then**

   
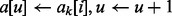

  **else**

   
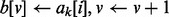

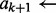
 the concatenation of *a* followed by *b*

To identify where maximal matches start, we need to keep track of the start position of matches between neighboring prefixes. Formally, for 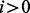 define 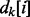 to be the smallest value *j* such that 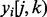 matches 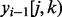 (note that I have dropped the *k* suffix of the *y*’s here and in the following for ease of notation, since its value is implicitly *k* for the time being). If 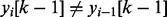 then set 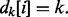 It can then be shown that the start of any maximal match ending at *k* between any 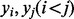 is given by 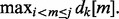 Using this we can efficiently extend Algorithm 1 to update *d_k_* in parallel with *a_k_* as we sweep through the data, as shown in Algorithm 2.

**Algorithm 2** BuildPrefixAndDivergenceArrays—build the divergence array 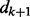 along with 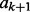 from *d_k_* and *a_k_*

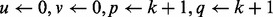

create empty arrays 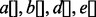

**for** 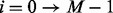 **do**

  **if** 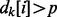 **then** 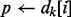

  **if** 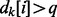 **then** 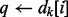

  **if** 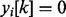 **then**

   
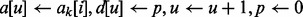

  **else**

   
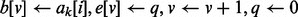

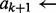
 the concatenation of *a* followed by *b*

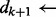
 the concatenation of *d* followed by *e*

Because we are dealing with bi-allelic data, so long as 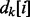 > 0, the values of 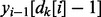 and 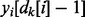 must be 0 and 1, respectively, since they differ by definition and are in sorted order. This means as a corollary that it is not possible for 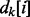 to be equal to 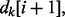 as long as they are greater than 0, because otherwise 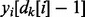 would need to be both 1 and 0, which is impossible.

I will call the collection of arrays *a_k_* for all *k* the ‘positional prefix arrays’ of *X*. These are related to standard suffix arrays, but apart from being prefix rather than suffix arrays because the sorting is in the reverse direction, they differ because they form a set of *N* arrays each of size *M* rather than a single array of size *NM*. The *d_k_* contain the equivalent of ‘longest common prefix’ values in standard suffix array algorithms.

### 2.2 Finding all matches within *X* longer than a minimum length *L*

Now we can use the *a_k_* and *d_k_* arrays to efficiently find matches. In order to count matches only once, we will sweep through the sequences with increasing *k* and only report matches at each *k* that end at *k*, i.e. for which 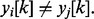 In order for the match to be longer than *L*, we must by definition have 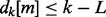 for all 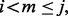 so pairs of indices (*i*, *j*) with this property will occur in blocks in the sorted list, separated by positions at which 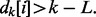 We therefore proceed through the sorted list at position *k*, keeping track of the last time that *d_k_*[*i*] was greater than (*k* − *L*). Given this, our algorithm looks remarkably like that for generating the *a_k_* arrays.

**Algorithm 3 ReportLongMatches—report matches within *X* ending at *k* longer than *L***

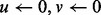
, create empty arrays 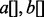

**for** 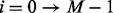 **do**

  **if** 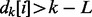 **then**

   **if** 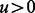 **and** 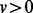 **then**

    **for all** 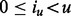 **and** 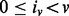 **do**

      report match from 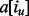 to 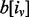 ending at *k*

   
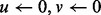

  **if** 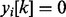 **then**

   
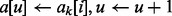

  **else**

   
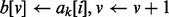

This algorithm is 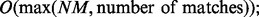 reporting the pairs of subsets *a*[] and *b*[] of sizes *u* and *v* would be *O*(*NM*). Note that ReportLongMatches can be run in the same sweep through the data as used to calculate the *a* and *d* arrays, so if we are happy to discard previous values of *a_j_* and *d_j_* for *j* < *k* as we go, it can be carried out in *O*(*M*) space.

A variation of this method can deliver all matches that extend in both directions from a location by at least a minimum length *L/*2. In this case one considers blocks within which 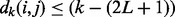 to find sets of such matches centered on position 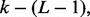 and does not separate into subsets for which *y_k_*[*i*] = 0 or 1. This approach may be relevant when looking for similar sequences at a position, perhaps for the purpose of imputation. Long matches will recur many times in this formulation, so it is best if possible to use the similar subsets as they are identified during the sweep, rather than to store them for future use.

### 2.3 Finding all set-maximal matches within *X* in linear time

Consider a sequence *y_i_* in the sorted list at *k*. Under what conditions will it have a set-maximal match ending at *k*? Clearly the match must be to one or more sequences directly preceding or following it in the sort order. First we find the candidate interval [*m*,*n*] such that for all 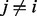 with 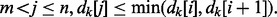 If 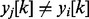 for all these *j* then *y_i_* has a set-maximal match to them all, but (unless *K* = *N*) if any have 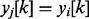 then the match between *y_i_* and *y_j_* can be extended forwards, and there is no set-maximal match ending at *k*. Iterating over all *k* and *i* we get algorithm 4.

**Algorithm 4 ReportSetMaximalMatches—report set maximal matches in *X***

**for** 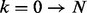 **do**

  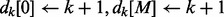 ▹ sentinels at boundaries

  **for** 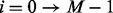 **do**

   
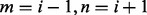

   **if** 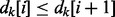 **then** ▹ scan down the array

     **while** 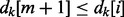 **do**

      **if** 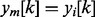 **and** 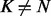 **next** i

      
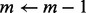

   **if** 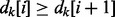 **then** ▹ scan up the array

     **while** 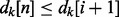 **do**

      **if** 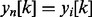 **and** 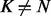 **then next** *i*

      
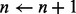

   **for** 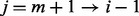 **do**

     report match of 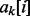 to 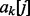 from 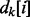 to *k*

   **for** 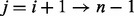 **do**

     report match of 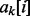 to 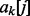 from 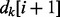 to *k*

Despite the inner loops this algorithm only has time complexity *O*(*NM*), because the requirement that 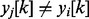 limits the search so that each position is compared at most once from each direction. To be completely precise, because matches have to terminate at the start and end of the sequence, this last statement relies on there not being arbitrarily large groups of sequences identical from 0 to *N*. Under these conditions, this also proves that the total number of set-maximal matches within *X* is bounded by a fixed multiple of *NM*.

As with ReportLongMatches, ReportSetMaximalMatches can be run in the same sweep through the data as used to calculate the *a* and *d* arrays, so if we are happy to discard previous values of *a_j_* and *d_j_* as we go, it also can be carried out in *O*(*M*) space.

### 2.4 Finding all set-maximal matches from a new sequence *z* to *X*

Next let us consider the case where we have a new sequence *z*, and want to find the set-maximal matches between it and set *X*. We will again sweep forward through the sequence, and in this case keep track of the start *e_k_* of the longest match of *z* to some *y_i_* ending at position *k*, and the interval 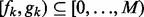 of indices in *a_k_* with that match. So for all *i* such that 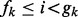 we have 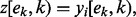 but 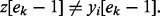 We allow *g_k_* to be *M* if 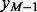 is included in the set of longest matches.

We want an efficient procedure for updating *e*, *f* and *g* as we move from *k* to (*k* + 1). First let us imagine that we have a procedure for updating *f* and *g* to *f′* and *g′* given a fixed starting position *e_k_*. If 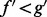 then at least some of the original matches starting at *e_k_* and ending at *k* can be extended to (*k *+ 1), so 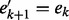 by definition and we are done. If on the other hand 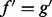 then none of the matches can be extended, and so the matches ending at *k* to sequences between *f_k_* and *g_k_* are set-maximal and can be reported. Then we need to find a new 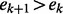 and corresponding new 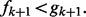


To efficiently update *f* and *g* we need the values *u* and *v* from Algorithm 1. We did not store them at the time, but let us now assume that we did so, in arrays *u_k_* and *v_k_*, and also kept track of the total number of zero values at position *k* in the sequences as value *c_k_*, which is equivalently the length of array *a*[] in Algorithm 1. Now if we define 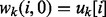 and 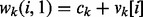 then Algorithm 1 tells us that 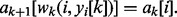 Furthermore, if 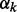 is the inverse of the permutation *a_k_*, then 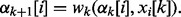 This last statement gives us a clue about how to update *f* and *g*. If we define 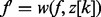 then this will be the index in 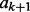 of the first sequence *y_j_* with 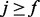 for which 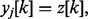 which is what want. Similarly 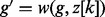 is what we want for updating *g*. So we can now update *f* and *g* by simple lookup from stored values.

Now, as we saw above, if 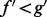 then we are done. On the other hand, if 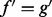 then there are no extensions of the match starting at *e_k_*. At position *k*, we know that *z* sorted either just before the block [*f*,*g*) in the natural prefix ordering, or just after it. So it either sorted just before *f* or just before *g*. From this we can infer that 
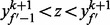
 in the natural ordering of reversed prefixes. So 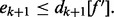 Let us consider 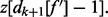 If this is 0 then *z* matches 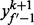 better than 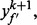 and we will set 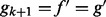 and look for 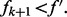 If it is 1 then *z* matches 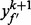 better than 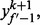 and we will set 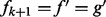 and look for 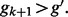 In either case we need to search back from 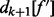 to find 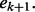 We need to take a little care at the boundaries 0 and *M*.

**Algorithm 5 UpdateZmatches—report any set-maximal matches of *z* to *X* ending at *k* and update to (*k* + 1)**

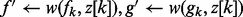

**if** 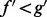 **then**

  
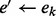

**else**

  **for** 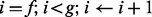 **do** report match to 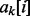 from *e* to *k*

  
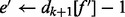

  **if** 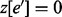 **and** 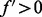 **then**

   
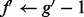

   **while** 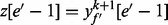 **do** 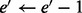

   **while** 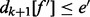 **do** 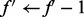

  **else**

   
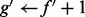

   **while** 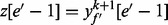 **do** 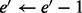

   **while** 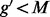 **and** 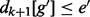 **do** 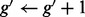

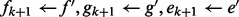

It is not immediately obvious that the algorithm is *O*(*N*). The while loop in *f′* or *g′* is inevitable because it only takes as many iterations as there are matches to report the next time that 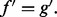 The total number of set-maximal matches is bounded by *NA*, as required. More complicated are the while loops that decrement *e′*. The sum of times these are used is bounded by a constant multiple of *N*.

### 2.5 Compact representation of *X*

The algorithms described above all use the *a_k_* and *d_k_* arrays to find matches. However, these are arrays of integers with a total number of elements equal to the number of binary values in the original dataset, so storing them would take more space than a bit representation of the starting data. Some algorithms can be applied on the fly, in the same sweep through the data as used to generate the values of *a* and *d*, but for other purposes, in particular for analyzing new sequences, we would like to store the relevant information more efficiently. Here is a description of how to do that.

First we notice that in the matching processes we do not actually use directly the 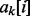 indexes, but rather the 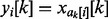 values. These are a permutation of the 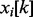 values determined by the *a_k_* permutation indicating the sort order at *k* of prefixes up to position (*k* − 1), and are therefore a positional analogue of the Burrows–Wheeler Transform (BWT) of *X* (Burrows and Wheeler, 1994; see Li and Durbin (2009) for an explanation closer to that given here if this is not familiar). Let us call the set of ordered *y* sequences the Positional Burrows–Wheeler Transform (PBWT). As with the BWT, the PBWT is composed of bit values not integer values, so we can store it in the same space as the original data. Furthermore we can also expect the *y* arrays to be strongly run-length compressible. This is because population genetic structure means that there is local correlation in values due to linkage disequilibrium, which means that haplotypes with similar prefixes in the sort order will tend to have the same allele values at the next position, giving rise to long runs of identical values in the *y* array. So the PBWT can easily be stored in smaller space than the original data. This will be true even if the original data is run-length encoded, since the left-to-right orientation of the data in *X* will not reflect shared haplotype structure due to linkage disequilibrium.

To find matches to a new sequence, as in Algorithm 5, we need also the stored arrays *u_k_* and *v_k_*, in order to evaluate the extension function 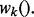 These arrays correspond to the information stored in the index described by Ferragina and Manzini (2000), commonly known as the FM-index. Given the PBWT we can store the information needed to generate them efficiently in an exactly analogous fashion to that for normal strings.

We do need values of 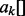 for reporting, but given the *y* values in the PBWT and the position FM-index, we can do this efficiently by only storing *a_k_* for a subset of values of *k*, for example every 32 or 64 positions. Reported matches will be longer than this, so extending to the next stored value of *a* by use of the extension function 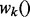 is relatively inexpensive.

Finally, we need a compact representation of the *d_k_* arrays. For now, it is proposed to Huffman encode the differences between adjacent 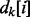, and perhaps only store them at a subset of *k* as for *a_k_*. There is probably scope for further improvement here.

## 3 RESULTS

Here I present initial results on simulated data. A dataset of 100 000 haplotype sequences covering a 20Mb section of genome sequence was simulated using the sequentially Markovian coalescent simulator MaCS Chen (2009) using essentially the command macs 100000 2e7 -t 0.001 -r 0.001 (in fact a larger simulation was undertaken, which crashed a little beyond 20Mb, and the remaining material was trimmed down to this set). There are 370 264 segregating sites in this dataset. The raw MaCS output contains essentially the haplotype sequences written in 0’s and 1’s, and so is approximately 37GB in size. This compresses with gzip to 1.02GB.

An initial implementation pbwt of the key algorithms was produced. This uses single byte run length encoding for the PBWT, with the top bit encoding the value, the next two bits selecting whether the length is in units of 1, 64 or 2048, and the remaining 5 bits giving the number of units. For runs >64 but <2048 this typically requires 2 bytes, and for runs >2048 but <64k this typically requires 3 bytes. All experiments were carried out on an Apple Mac Air laptop with a 2.13GHz Intel Core 2 Duo processor using a single core. Encoding the dataset of 100 000 sequences described above took 1070 s (user plus system), generating a PBWT representation that is 7.7MB in size, over 130 times smaller than the gzip compression of the raw data. Further results including application to subsets of the data are given in Table 1, showing that the relative gain increases with the number of sequences, indicating clearly the non-linear benefits of the algorithm. This can be clearly seen by the observation that for each increase of a factor of ten in the number of sequences, the average number of bytes used by the PBWT to store the haplotype values at a site only approximately doubles. As a test on real data, similar measures were applied to the chromosome 1 data from the 1000 Genomes Project phase 1 data release 1000 Genomes Project (2012), consisting of 2184 haplotypes at 3 007 196 sites. The gzip file of this data took 303MB, whereas the PBWT used 51.1MB, nearly a factor of six smaller, not far from the factor expected based on the simulated data.

**Table 1.** Compression performance of pbwt on datasets of increasing size

Number of sequences	1000	10 000	100 000
Sequences .gz size (KB)	10 515	105 559	1 024 614
PBWT size (KB)	1686	3372	7698
Ratio .gz/PBWT	6.2	31.3	133.1
PBWT bytes/site	4.6	9.1	20.8

Next Algorithm 4 was implemented to find all set-maximal matches within the simulated datasets. As expected the time taken was linear in the number of sequences (Table 2), taking only 20 min to find all maximal shared substrings within 100 000 sequences.

**Table 2.** Set-maximal match performance of pbwt on datasets of increasing size

Number of sequences	1000	10 000	100 000
Set-maximal time (s)	12.1	120.3	1213.7
Set-maximal average length (Mb)	0.27	1.48	3.98

Finally the comparative performance of three different approaches to matching new sequences to a pre-indexed reference panel was evaluated, finding all set-maximal matches of each new sequence to the reference set. For this evaluation, I subsampled the simulated sequence dataset to approximate typical data from a genotype array experiment, by only retaining a fraction (10%) of sites with allele frequency >5%. This reduced the number of sites to 5940, approximately one pre 3.4 kb, corresponding to 850 k in the human genome, comparable to the content of a standard a genotyping array. Table 3 shows results for matching 1000 of the sequences from the simulation, comparing them to non-overlapping subsets between 1000 and 50 000 in size.

**Table 3.** Time to match 1000 new sequences in seconds, split into user (u) and system (s) contributions for the indexed and batch approaches

Number of sequences	1000	5000	10 000	50 000
Naïve	52.1	258.9	519.2	2582.6
Indexed	0.9u + 0.1s	0.9u + 0.1s	0.9u + 0.2s	1.7u + 15s
Batch	2.3u + 0.1s	3.5u + 0.1s	4.8u + 0.1s	12.1u + 0.1s

First a ‘naive' algorithm was implemented, in which each sequence was compared to all sequences in the panel in a single pass, keeping the best matching segment covering each base in the test sequence. As expected, this approach takes time linear in the size of the panel, taking ∼0.05 s per sequence in the panel. Second Algorithm 5 was implemented, which is termed ‘indexed’ in Table 3. This takes constant user time of 0.9 user seconds to match 1000 sequences to reference panels up to size 10 000, but for 50 000 reference sequences the size of the stored *u*, *v* and *d* arrays (which were not compressed in this implementation) became larger than the available memory, resulting in an increase in system time from 0.2 to 15 s, and a smaller increase in user time to 1.7 s. I therefore conclude that the PBWT-based approach can be hundreds of times faster than a direct search approach and find matches in time independent of the reference panel size, as conjectured above, so long as the associated index arrays fit in memory.

For situations where the indexes do not fit in memory, we can still use the PBWT data structures to provide a third ‘batch’ matching process that is still much faster than the naive approach. This uses a modified version of the within-set Algorithm 4 that passes jointly through the panel and combined set of new sequences together, just considering matches between new and old sequences. As shown in Table 3, the time taken by this batch approach, whose memory requirements are low and independent of the number of sites, increases with reference panel size, but is still many times more efficient than a direct search as in the naive approach. Asymptotically the time taken by current implementation will depend linearly *M*, but it may be possible to reduce this by careful avoidance of PBWT compression where it is not needed.

## 4 DISCUSSION

I have presented here a series of algorithms to generate positional prefix array data structures from haplotype sequences and to use them for very strong compression of haplotype data, and time and space efficient haplotype matching. In particular, the matching algorithms remove a factor of *M*, the size of the set of haplotype sequences being matched to, from the search time taken by direct pair-wise comparison methods. This makes it possible to find all best matches within tens of thousands of sequences in minutes, and generates the potential for practical software that scales to millions of sequences. Although the algorithms are presented for binary data, they can be extended to multi-allelic data with a little care.

These algorithms share aspects of their design with analogous algorithms based on suffix arrays for general string matching, but are structured by position along the string resulting in substantial differences. One consequence is that, unlike with suffix array methods where linear time sorting algorithms are non-trivial, building the sorted positional prefix arrays in linear time using Algorithm 1 is straightforward. The approach used here is reminiscent of that used by Bauer *et al.* (2011) to generate a string BWT from very large sets of short strings.

With respect to efficient representation, it is interesting to note that the original BWT was introduced by Burrows and Wheeler (1994) for string data compression, not search, and it in fact forms the basis of the bzip compression algorithm. Valimaki *et al.* (2007) have previously explored the use of BWT compressed self-index methods for efficient compression and search of genetic sequence data from many individuals, but this does not require a fixed alignment of variable sites as in the work presented here, and is substantially different.

All the algorithms described here require exact matching without errors or missing data. As for sequence matching, if a more sensitive search is required that permits errors, it is still possible to use the exact match algorithms to find seed matches, and then join or extend these by direct testing. This would typically be the approach taken by production software, but having powerful methods to identify seeds is key to performance.

An alternative to using suffix/prefix array methods in sequence matching is to build a hash table to identify exact seed matches. Analogous to the creation of position prefix arrays described here, it would be possible to build a set of positional hash tables for each position in the haplotype sequences. Hash based methods when well tuned can be faster than suffix array based methods, because the basic operations are simpler, but they typically require greater memory, particularly in cases where the suffix representation can be compressed as it can be here. A problem with genotype data not present in standard sequence matching is that the information content of positions varies widely, with a preponderance of rare sites with very little information, which would mean that the length of hash word would need to change depending on position in the sequence. An alternative would be to build hashes based on a subset of sites with allele frequency greater than some value such as 10%, or in some frequency range, but this would lose information leading to false seed matches.

Most research into algorithms for analyzing large sets of haplotype or genotype data has focused on statistical methods that are powerful for inference, but only scale up to a few thousand sites and sequences; e.g. see Marchini and Howie (2010). Recently accelerated methods have been developed that can handle data up to tens of thousands of sequences, e.g. Williams *et al.* (2012) or Delaneau *et al.* (2012). However, these methods provide approximations to the statistical matching approaches, and are still much heavier than the algorithms presented here. Over a million people have been genotyped, and although there are logistical issues in bringing together datasets on that scale, genotype data on sets of >100 000 people are becoming available (Hoffman *et al.*, 2011). One approach to more efficient phasing and imputation may be to use computationally efficient approaches such as the positional prefix array methods to seed matches for statistical genotype algorithms, or at other computational bottlenecks. For example, in their BEAGLE software Browning and Browning (2007) build a probabilistic hidden Markov Model from a variable length Markov model of the local haplotype sequences which is essentially derived from a dynamic truncation of the positional prefix array. Although their algorithms using this probabilistic model take them in a different direction, I suggest that the methods described here could be used to significantly speed up the model building phase of BEAGLE.

Alternatively, a more direct approach may also be possible. Most phasing and imputation algorithms build a model from the entire dataset, then thread each sequence in turn against it to provide a new phasing based effectively on a series of matches. Instead, the positional prefix algorithms progress jointly along all sequences. If we start at both ends of the data, then at some position *k* we have information about matches in both directions based on the current phasing, and can propose an assignment of alleles for all sequences at *k* in a single step, before incrementing *k*. Approaches based on this idea may be fast and complementary to current methods.
